# Microstructure Engineered Nanoporous Copper for Enhanced Catalytic Degradation of Organic Pollutants in Wastewater

**DOI:** 10.3390/ma18132929

**Published:** 2025-06-20

**Authors:** Taskeen Zahra, Saleem Abbas, Junfei Ou, Tuti Mariana Lim, Aumber Abbas

**Affiliations:** 1 School of Materials Engineering, Jiangsu University of Technology, Changzhou 213001, China; taskeen.zahra5@outlook.com (T.Z.); oujunfei_1982@163.com (J.O.); 2 School of Mechanical Engineering, Shandong University of Technology, Zibo 255049, China; dr.saleem.abbas@outlook.com; 3 School of Civil and Environmental Engineering, Nanyang Technological University, Singapore 226019, Singapore

**Keywords:** nanoporous copper, microstructure, catalytic degradation, organic pollutants, methylene blue

## Abstract

Advanced oxidation processes offer bright potential for eliminating organic pollutants from wastewater, where the development of efficient catalysts revolves around deep understanding of the microstructure–property–performance relationship. In this study, we explore how microstructural engineering influences the catalytic performance of nanoporous copper (NPC) in degrading organic contaminants. By systematically tailoring the NPC microstructure, we achieve tunable three-dimensional porous architectures with nanoscale pores and macroscopic grains. This results in a homogeneous, bicontinuous pore–ligament network that is crucial for the oxidative degradation of the model pollutant methylene blue in the presence of hydrogen peroxide. The catalytic efficiency is assessed using ultraviolet–visible spectroscopy, which reveals first-order degradation kinetics with a rate constant *κ* = 44 × 10^−3^ min^−1^, a 30-fold improvement over bulk copper foil, and a fourfold increase over copper nanoparticles. The superior performance is attributed to the high surface area, abundant active sites, and multiscale porosity of NPC. Additionally, the high step-edge density, nanoscale curvature, and enhanced crystallinity contribute to the catalyst’s remarkable stability and reactivity. This study not only provides insights into microstructure–property–performance relationships in nanoporous catalysts but also offers an effective strategy for designing efficient and scalable materials for wastewater treatment and environmental applications.

## 1. Introduction

The development of nanoporous structured materials has emerged as a pivotal area in materials chemistry due to their remarkable physical, mechanical, and chemical properties [1,2,3]. Their intrinsic characteristics—including nanoscale effects, large specific surface areas, and low densities—grant them superior catalytic activity and broad applicability in fields such as sensing, actuation, and heterogeneous catalysis, particularly for organic oxidation reactions [4,5,6,7]. Among pressing environmental concerns, the release of synthetic dyes into water bodies—primarily from textiles, paper, and food industries—poses significant ecological and health risks. Methylene blue (MB), a widely used cationic dye, is notably resistant to natural degradation and presents persistent contamination in wastewater [8,9]. Hence, the efficient and complete degradation of such organic dyes into non-toxic end products is critically needed [8,10,11,12,13,14,15]. Nanoporous metals offer a promising pathway, as their highly porous structures provide abundant active sites for oxidative degradation reactions.

Recent studies have explored diverse materials for the removal of MB from wastewater, including metal oxides [8], clay-based composites [9], biochar-supported catalysts [11], and non-thermal plasma techniques [12]. For example, Karim et al. reported the Fenton-like degradation of MB using iron-oxide loaded attapulgite clay composites with >90% efficiency under ambient conditions [8], while Srivastava et al. demonstrated MB degradation via CuO nanostructures synthesized through methanol-assisted methods, achieving ~60% removal over 240 min [16]. Such works highlight the potential and limitations of conventional systems and motivate the development of more structurally optimized catalysts for rapid and complete pollutant degradation.

Among others [17,18,19], nanoporous copper (NPC) stands out for its desirable attributes: excellent chemical and thermal stability, high conductivity, electrocatalytic activity, and low material cost [20,21]. Recent studies have highlighted the effectiveness of copper-based catalysts for the oxidative degradation of MB in the presence of hydrogen peroxide (H_2_O_2_) [22,23,24,25]. While nanoparticles and copper oxides have shown catalytic activity, nanoporous copper’s interconnected porous network and tunable surface morphology offer potential for enhanced performance. Despite these advances, the role of microstructural features in influencing the catalytic efficiency of NPC has not been thoroughly investigated. Since catalytic activity is intimately linked to structural properties, understanding the structure–property–performance relationship is crucial for rational catalyst design [26,27,28,29].

Among the various fabrication methods—such as hydrothermal synthesis, templating, and dealloying—chemical dealloying is especially promising due to its simplicity and ability to generate three-dimensional, bicontinuous porous architectures [30,31,32,33,34,35]. The dealloying process involves selective dissolution of the more electrochemically active element from an alloy, typically yielding a network of nanoscale ligaments and pores. However, factors such as the initial alloy composition and microstructure play a critical role in determining the final morphology and functionality of the resulting NPC [36,37,38,39,40,41,42,43,44]. Particularly, amorphous precursor alloys offer superior structural uniformity due to their homogeneous composition, facilitating the formation of defect-free NPCs. Yet, studies exploring the relationship between alloy composition, dealloying behavior, and catalytic performance remain limited.

In this study, we systematically investigate the microstructure-engineered fabrication of NPC using amorphous alloy precursors and assess the influence of porous morphology on its catalytic activity for MB degradation. NPC samples featuring bicontinuous, interpenetrating ligament–channel structures were synthesized through controlled chemical dealloying of Ti_30_Cu_70_ and Ti_40_Cu_60_ alloy ribbons. The resulting variations in pore geometry and ligament architecture were correlated with catalytic efficiency under oxidative conditions in the presence of hydrogen peroxide. Detailed kinetic studies were conducted to quantify degradation performance, and a mechanistic pathway for MB oxidation over NPC surfaces is proposed. This study provides critical insights into the microstructure’s role in enhancing catalytic performance and contributes to the rational design of nanoporous materials for advanced wastewater treatment applications.

## 2. Experimental

### 2.1. Materials

**Figure 1:**
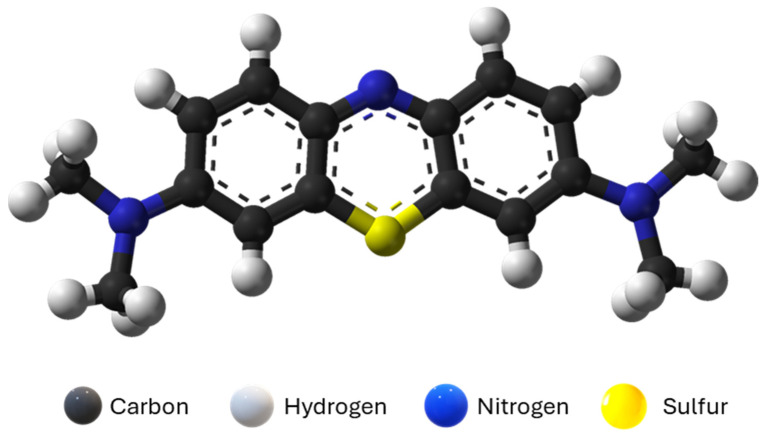
Three-dimensional chemical structure of MB dye [45].

High-purity copper (Cu) and titanium (Ti) metals (99.9%) were procured from AMERICAN ELEMENTS^®^, Los Angeles, CA, USA. Methylene blue (C_16_H_18_ClN_3_S) was employed as a model organic dye for catalytic degradation studies (see Figure 1). Hydrofluoric acid (HF), hydrogen peroxide (H_2_O_2_, 30 wt.%), and all other reagents were of analytical grade and were sourced from local suppliers. Ultrapure water with a resistivity of 18.2 MΩ·cm was used throughout all experiments to ensure consistency and purity in sample preparation and analysis.

### 2.2. Synthesis of Nanoporous Copper

Button-shaped ingots of Ti_x_Cu_(100−x)_ (x = 30 and 40 at.%) alloys were first prepared by arc melting high-purity Ti and Cu metals in an argon atmosphere. The resulting alloy buttons had a diameter of 20 mm and a thickness of approximately 5 mm. To obtain rapidly solidified ribbons suitable for dealloying, the ingots were remelted using a high-frequency induction furnace under an inert argon atmosphere and processed via melt-spinning onto a single copper roller chilled with circulating water. This yielded amorphous ribbons with a thickness of 20–30 µm, width of 2–3 mm, and lengths extending to several centimeters. Chemical dealloying of the Ti_30_Cu_70_ and Ti_40_Cu_60_ ribbons was carried out in 0.13 M HF solution at ambient temperature. A fixed dealloying duration of 3 h was employed for all samples based on prior optimization to ensure the complete removal of Ti and to obtain structurally stable and reproducible NPC frameworks. After 3 h of dealloying, the samples were retrieved, thoroughly rinsed with ethanol to remove residual HF and metal ions, and subsequently stored in ethanol to prevent oxidation. The resulting nanoporous copper samples achieved after dealloying Ti_30_Cu_70_ and Ti_40_Cu_60_ ribbons were designated as NPC-1 and NPC-2, respectively.

### 2.3. MB Degradation

The catalytic degradation of MB was conducted in a 50 mL sealed flask under ambient conditions. In a typical experiment, 10 mL of MB solution (10 ppm), 2 mL of hydrogen peroxide (30 wt.%), and 0.01 g of the NPC catalyst were mixed and stirred at a constant speed. The flask was sealed throughout the reaction to prevent solvent evaporation. At designated time intervals, small aliquots of the reaction mixture were extracted using a dropper pipette and transferred to quartz cuvettes for analysis. Samples were collected at 10–15 min intervals, with more frequent sampling (every 10 min) during the early stages of degradation to accurately capture reaction kinetics. Ultraviolet–visible (UV–vis) spectroscopy was performed using an Evolution 220 spectrophotometer (Thermo Scientific, Waltham, MA, USA) to monitor the degradation of MB by measuring the absorbance peak at 664 nm. To maintain the solution volume and minimize error, each sample was promptly returned to the reaction flask within 30 s after measurement. MB concentration was quantified based on Beer–Lambert’s law using a pre-established linear calibration curve relating absorbance intensity at 664 nm to dye concentration [46].

### 2.4. Catalyst Characterization

To investigate the structural and morphological properties of the synthesized NPC catalysts, a series of characterization techniques were employed. X-ray diffraction (XRD) analysis was performed using an X’Pert Pro MPD diffractometer (PANalytical, Tokyo, Japan) to identify crystalline phases and assess the crystallinity of the samples. The measurements were carried out using Cu Kα radiation (λ = 1.5406 Å) over a 2θ range of 20° to 80°, with a scan rate of 0.02°/s. Field emission scanning electron microscopy (FESEM, SU-70, Hitachi, Tokyo, Japan) was used to visualize the surface morphology and evaluate pore–ligament architecture. Samples were sputter-coated with gold to improve conductivity prior to imaging. Transmission electron microscopy (TEM) and high-resolution TEM (HR-TEM) were carried out using a JEM-2100 microscope (JEOL, Tokyo, Japan) operated at 200 kV to examine the internal microstructure, crystallite size, and lattice fringes at the nanoscale. Selected area electron diffraction (SAED) patterns were obtained to confirm the crystallographic phases present in the NPC framework. These combined techniques provided comprehensive information on the phase, morphology, and microstructural evolution of NPC, which were critical to understanding the structure–property relationship governing catalytic activity.

## 3. Results and Discussion

### 3.1. Structure and Morphology

**Figure 2:**
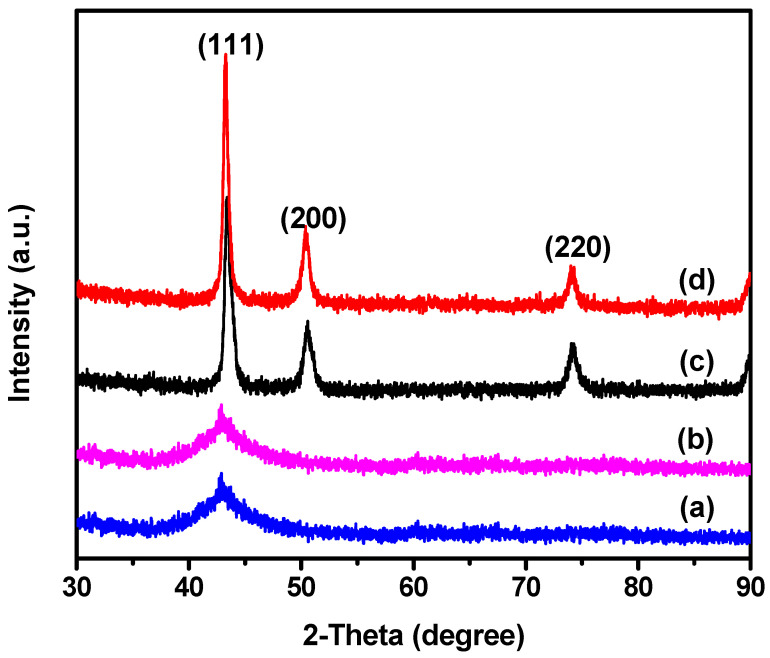
XRD patterns of as-prepared melt-spun Ti_30_Cu_70_ alloy ribbon (a), melt-spun Ti_40_Cu_60_ alloy ribbon (b) before dealloying, and corresponding NPC-1 (c), and NPC-2 (d) obtained after dealloying the alloy ribbons, respectively.

NPC was synthesized through a controlled chemical dealloying process, utilizing rapidly solidified amorphous Cu–Ti alloy ribbons as precursors. The two compositions of Ti_30_Cu_70_ and Ti_40_Cu_60,_ which are essential for producing uniform nanoporous copper structures upon dealloying, were focused on due to their demonstrated ability to form homogeneous amorphous alloy ribbons through melt spinning. These two compositions were selected to systematically investigate the influence of precursor alloy microstructure on the resulting pore morphology and catalytic performance. The synthesis involved a two-step procedure: first, the preparation of homogeneous amorphous alloy ribbons, and second, selective dissolution of the less noble element (Ti) from the Ti–Cu alloy ribbons to yield a three-dimensional nanoporous copper structure. The reaction proceeds as Ti + 6HF → H_2_TiF_6_ + H_2_↑, where titanium is converted into soluble hexafluorotitanic acid (H_2_TiF_6_) and hydrogen (H_2_) gas is released, as evidenced by continuous bubbling during the reaction. To investigate the structural evolution before and after dealloying, XRD was employed for phase identification and crystallinity assessment. As shown in Figure 2 (two bottom patterns), the as-prepared Ti_30_Cu_70_ and Ti_40_Cu_60_ alloy precursors exhibited broad and featureless diffraction humps centered around 43°, which is characteristic of amorphous metallic phases. The absence of sharp Bragg peaks confirms the non-crystalline nature of the initial alloy ribbons. Following chemical dealloying of Ti_30_Cu_70_ and Ti_40_Cu_60_ alloy ribbons in hydrofluoric acid, NPC-1 and NPC-2 were obtained, respectively, showing a distinct transformation in the XRD patterns (Figure 2, two top patterns). The NPC samples obtained exhibited sharp and well-defined peaks at 43.4°, 50.4°, and 73.4°, which correspond to the (111), (200), and (220) crystal planes of the face-centered cubic (FCC) structure of metallic copper (Cu^0^) (JCPDS No. 04-0836) [47]. The appearance of these peaks confirms the formation of crystalline copper as a result of the selective dissolution of titanium from the alloy matrix. The increased intensity and sharpness of these reflections further indicate that the resulting NPC possesses a high degree of crystallinity. This structural evolution from an amorphous precursor to a highly crystalline nanoporous copper framework is essential, as the crystallinity and pore geometry can significantly influence the surface reactivity and catalytic behavior of the material. Although the completeness of dealloying was confirmed by XRD analysis in this study, future investigations should incorporate EDX elemental mapping to further confirm the removal of titanium and to assess the trace elemental distribution within the nanoporous framework. Additionally, future XPS and ICP analyses would be valuable for validating the oxidation state and quantifying elemental composition.

**Figure 3:**
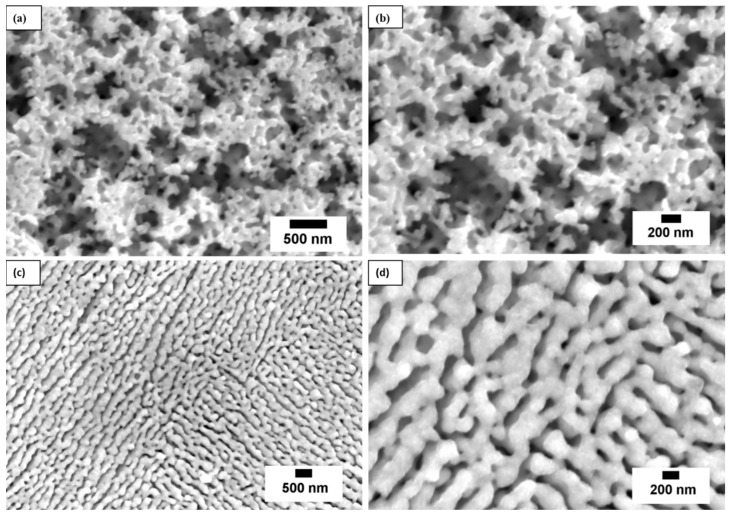
(**a**,**b**) Low- and high-resolution SEM images of NPC-1 and (**c**,**d**) NPC-2, showing bicontinuous pore–ligament structure.

The microstructural morphology of the synthesized NPC was examined using field emission scanning electron microscopy (FESEM). Figure 3 displays representative SEM micrographs highlighting the influence of initial alloy composition on the resulting porous architecture after chemical dealloying. The NPC-1 exhibited a highly porous structure with a broad pore size distribution ranging from approximately 50 nm to 300 nm (Figure 3a,b). The pores appeared irregular and inhomogeneous, suggesting a less controlled dealloying process. The relatively low titanium content (30 at.%) in the precursor alloy may have led to uneven dissolution kinetics, resulting in spatial variations in pore growth direction and size. This inconsistency in pore morphology could compromise structural uniformity and, consequently, catalytic efficiency.

In contrast, the NPC-2 exhibited a significantly more uniform morphology, as shown in Figure 3c,d. A well-defined bicontinuous interpenetrating ligament–channel network was observed, with an average pore diameter of ~100 nm and ligament width of ~200 nm. The uniformity in structure can be attributed to the higher titanium content, which promotes more consistent dissolution and pore propagation throughout the alloy matrix. Remarkably, no cracks, voids, or other structural defects were visible, indicating the high mechanical integrity and quality of the NPC. These findings underscore the critical role of precursor composition in tailoring the microstructure of NPC, which in turn influences its functional properties. The formation of a homogenous, defect-free porous network is essential for maximizing surface area and enhancing catalytic performance. Although precise BET surface area and volume porosity measurements were not conducted in this study, SEM and TEM imaging suggest a highly porous framework with estimated porosity in the range of 60–75% (based on pore diameter of ~100 nm and ligament width of ~200 nm), consistent with previous reports on nanoporous copper fabricated under similar conditions [36,37,38,48,49]. Future works should incorporate quantitative surface area analysis to further support structure–activity correlations.

**Figure 4:**
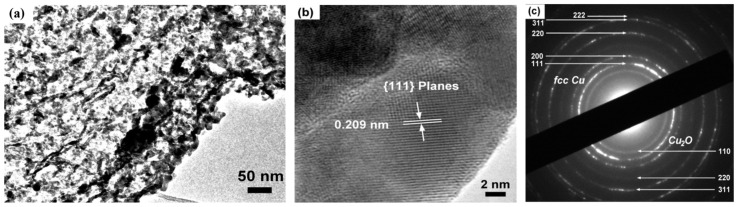
(**a**) Bright field TEM image, (**b**) high resolution TEM (HR-TEM) image, and (**c**) SAED pattern of NPC-2. The Cu_2_O rings observed in the SAED pattern are attributed to minor oxidation during sample preparation.

To gain deeper insight into the internal microstructure and crystallinity of the NPC, transmission electron microscopy (TEM) was performed on the NPC-2 sample. Figure 4 presents a bright-field TEM image, a high-resolution TEM (HR-TEM) image, and the corresponding selected area electron diffraction (SAED) pattern. The bright-field TEM image (Figure 4a) clearly reveals the nanoporous architecture of the material, with an average pore diameter of approximately 70 nm observed in the inner regions of the dealloyed ribbons. For this analysis, the ribbons were manually fractured to expose the internal structure. Notably, the pore sizes observed by TEM were smaller than those recorded in SEM, indicating a finer porous structure within the ribbon core, which may be attributed to the compositional and structural gradients developed during rapid solidification and dealloying. The smaller pore features observed in the bright-field TEM image (Figure 4a) compared to SEM (Figure 3) likely reflect the finer nanoporous structure within the interior regions of the dealloyed ribbons. This may be attributed to the compositional and structural gradients developed during rapid solidification and dealloying. The HR-TEM image (Figure 4b) confirms the face-centered cubic (FCC) crystal structure of the NPC. Lattice fringes are clearly visible, and the measured interplanar spacing of 0.209 nm corresponds to the (111) plane of FCC Cu. The presence of well-defined fringes signifies high crystallinity, and the observed polycrystalline texture suggests that copper atoms diffused and reassembled into ordered domains during the dealloying process. The SAED pattern (Figure 4c) further supports these observations. The upper diffraction rings correspond to the (111), (200), (220), and (311) planes of FCC Cu [50]. Interestingly, weaker diffraction rings in the lower region were indexed to the (110), (220), and (311) planes of Cu_2_O, indicating the presence of minor surface oxidation, likely introduced during sample handling and exposure to air. However, the low intensity of these Cu_2_O rings implies a minimal degree of oxidation.

Notably, the Cu_2_O peaks were not detected in the XRD patterns, which is attributed to the use of freshly prepared samples for XRD measurements of NPC-2 confirming the high purity of the as-prepared NPC. While XRD and SAED confirm the formation of metallic FCC copper with only minor surface oxidation, future work will incorporate XPS and ICP analyses to confirm the oxidation state and quantify elemental composition more precisely. These TEM results confirm the formation of a well-defined, nanoporous, and crystalline copper framework with minimal oxidation structural features that are essential for high-performance catalytic applications.

### 3.2. Oxidative Catalytic Reactivity

**Figure 5:**
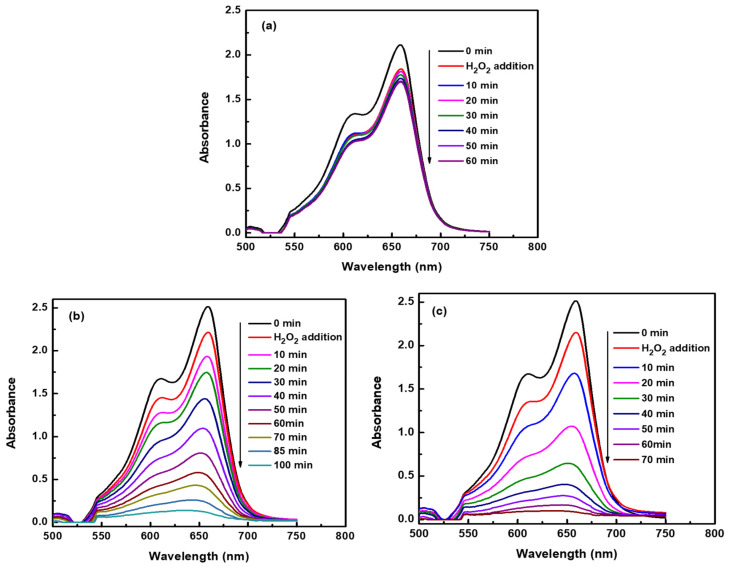
UV-vis adsorption spectra of MB degradation (**a**) without any catalyst (initial peak drop is due to dilution upon the addition of H_2_O_2_); (**b**) catalyzed with NPC-1 and (**c**) catalyzed with NPC-2.

The oxidative catalytic performance of NPC was evaluated through the degradation of MB in aqueous solution in the presence of H_2_O_2_. Time-resolved UV–visible spectroscopy was employed to monitor the degradation kinetics and assess the reactivity of the catalysts under ambient conditions. To establish a baseline, a control experiment was conducted by adding H_2_O_2_ to the MB solution without any catalyst (Figure 5a). A prominent absorption peak at 664 nm, characteristic of MB, was observed and remained largely unchanged over 60 min, indicating negligible degradation. This confirmed that H_2_O_2_ alone, under the given conditions, had limited oxidative capacity for MB degradation in the absence of a catalyst. In contrast, the addition of NPC catalysts significantly enhanced the degradation process. As shown in Figure 5b,c, both NPC-1 and NPC-2 exhibited strong catalytic activity, evident from the rapid decrease in the 664 nm absorption peak over time. For NPC-1, derived from the Ti_30_Cu_70_ precursor, the degradation of MB proceeded steadily, achieving near-complete decolorization within 110 min. The performance was markedly improved with NPC-2 (from Ti_40_Cu_60_), which facilitated nearly full degradation within just 70 min, highlighting its superior catalytic efficiency. The enhanced reactivity of NPC-2 can be attributed to its more uniform and well-connected pore–ligament microstructure, which likely provides a greater density of active sites and facilitates more effective electron transfer processes. These results demonstrate that NPCs not only serve as effective catalysts for the activation of H_2_O_2_ but also significantly accelerate the oxidative degradation of persistent organic dyes such as MB, offering a promising route for advanced wastewater treatment.

**Figure 6:**
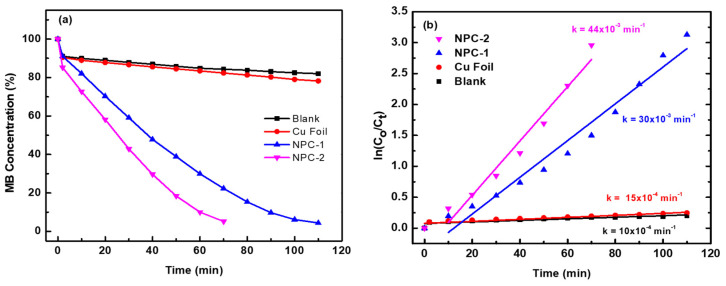
(**a**) The plot of MB degradation percentage vs. reaction time in the presence or absence of different catalysts. (**b**) First-order kinetic plot of ln(*C*_0_/*C_t_*) vs. time of MB degradation in the presence of different catalysts and H_2_O_2_ (2 mL, 30 wt.%) at ambient conditions.

To quantitatively assess the degradation performance, Figure 6a presents the percentage of MB degradation as a function of reaction time for the control group, bulk copper foil, and the as-prepared nanoporous copper (NPC) catalysts. In the absence of any catalyst, only ~3% of MB was degraded after 110 min in the presence of hydrogen peroxide, demonstrating the limited oxidative power of H_2_O_2_ alone. When bulk copper foil was introduced as the catalyst under identical conditions, the degradation efficiency showed a marginal increase to less than 5%, indicating that metallic Cu without a nanoporous structure lacks significant catalytic activity for MB degradation. In stark contrast, the nanoporous copper catalysts exhibited dramatically enhanced degradation performance. With NPC-1, near-complete degradation was achieved within 110 min, while NPC-2 demonstrated ultrafast catalytic efficiency, achieving full degradation in just 70 min. This superior performance is attributed to NPC-2’s homogeneous pore distribution and higher specific surface area, which provide abundant active sites for the catalytic activation of H_2_O_2_ and interaction with MB molecules.

The kinetics of MB degradation were further analyzed using the pseudo-first-order model, commonly applied for dye degradation processes. The reaction kinetics were modeled by the following equation:

(1) $$\ln\left(\frac{Co}{Ct}\right)=kt$$

where *C*_0_ and *C_t_* are the initial and time-dependent concentrations of MB, respectively, and *κ* is the apparent rate constant. The corresponding kinetic plots—ln(*C*_0_/*C_t_*) versus time—are presented in Figure 6b for all experimental conditions. The linearity of these plots confirms that the degradation follows pseudo-first-order kinetics. The rate constants *κ* were extracted from the slopes of the linear fits, yielding values of *κ* = 30 × 10^−3^ min^−1^ for NPC-1 and *κ* = 44 × 10^−3^ min^−1^ for NPC-2. These rate constants are substantially higher than those reported for comparable systems, such as copper oxide catalysts synthesized via methanol routes (κ = 13 × 10^−3^ min^−1^) [51] and micron-sized hollow copper spheres [52], emphasizing the superior reactivity of the microstructure-engineered NPC. The results clearly demonstrate the advantage of structural uniformity and nanoscale porosity in enhancing catalytic activity for oxidative degradation reactions.

These results clearly demonstrate that varying the composition of the Cu–Ti precursor alloys directly influences the microstructure of the resulting nanoporous copper, which in turn dictates its catalytic reactivity toward MB degradation. As shown in Figure 6b, the rate constant for MB degradation in the absence of any catalyst is only 10 × 10^−4^ min^−1^, while that for bulk Cu foil remains comparably low at 15 × 10^−4^ min^−1^, confirming their minimal contribution to the oxidative process. In sharp contrast, NPC-1 and particularly NPC-2 exhibit significantly higher rate constants, with NPC-2 outperforming all tested samples. The ultrafast catalytic activity of NPC-2 can be attributed to several synergistic factors. First, its interpenetrating bicontinuous ligament–channel structure imparts a substantially higher surface area, increasing the availability of active sites for the adsorption and degradation of dye molecules. Second, the nanostructured morphology introduces a dense network of edges, steps, and curved surfaces that are rich in catalytically active sites, which promote surface reactions [53]. Third, the inherent high electrical conductivity of metallic copper facilitates efficient electron transfer, accelerating the decomposition of hydrogen peroxide into highly reactive hydroxyl radicals (•OH). These radicals play a crucial role in the oxidative breakdown of MB, and their rapid generation is essential for enhanced catalytic performance [54]. Collectively, these structural and physicochemical features synergistically contribute to the superior degradation efficiency of NPC-2, establishing it as a highly effective catalyst for advanced wastewater treatment.

A comparative analysis of our NPC catalysts with various copper-based catalysts previously reported in the literature for MB degradation under similar experimental conditions is summarized in Table 1. Both the degradation time and the corresponding rate constants indicate that the catalytic performance of NPC, particularly NPC-2, is significantly superior. For instance, the degradation of MB using plate-like CuO catalysts has been reported to require up to 10 h [55], which is more than eight times longer than the 70 min observed for NPC-2. This extended degradation time corresponds to a notably lower rate constant of 3.7 × 10^−3^ min^−1^, approximately twelve times lower than the value achieved by NPC-2 (44 × 10^−3^ min^−1^). Similarly, Srivastava et al. reported MB degradation using CuO nanostructures synthesized via a methanol-assisted route, which required 240 min for completion, about four times longer than the reaction time for NPC-2 [16]. These comparisons clearly highlight the exceptional catalytic activity and efficiency of our microstructure-engineered nanoporous copper, underscoring its potential for the rapid and scalable oxidative degradation of organic pollutants in wastewater treatment applications.

### 3.3. Apparent Activation Energy

**Figure 7:**
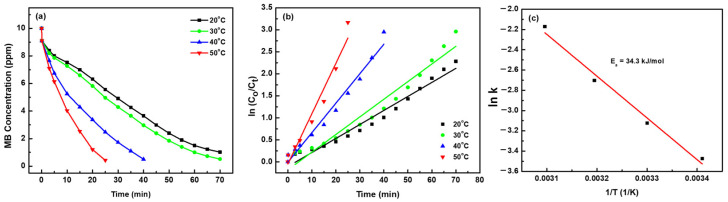
(**a**) The plot of MB concentration vs. reaction time at different temperature, (**b**) natural logarithm of MB concentration vs. reaction time at different temperature, (**c**) natural logarithm of rate constant vs. reciprocal of reaction temperature.

To gain deeper insight into the catalytic performance of NPC-2, the apparent activation energy (*E_a_*) was evaluated by conducting MB degradation experiments at different reaction temperatures. Determining *E_a_* provides a fundamental understanding of the reaction kinetics and energy barrier associated with the catalytic process. MB degradation in the presence of H_2_O_2_ and NPC-2 was carried out at 20 °C, 30 °C, 40 °C, and 50 °C, as shown in Figure 7a. An increase in reaction temperature led to a significant enhancement in the degradation rate. At 20 °C, approximately 90% of MB was degraded within 70 min, while at 50 °C, over 96% degradation was achieved in just 25 min.

This temperature-dependent acceleration in MB degradation can be attributed to the more rapid decomposition of H_2_O_2_ into highly reactive hydroxyl radicals (∙OH) at elevated temperatures [64], which are primarily responsible for the oxidative breakdown of MB. The degradation kinetics at each temperature were further analyzed using the pseudo-first-order rate model. As illustrated in Figure 7b, plots of ln(C_0_/C_t_) versus reaction time showed excellent linearity across all temperatures, confirming the applicability of first-order kinetics. From the slopes of these plots, the rate constants (κ) were calculated as 0.032 min^−1^, 0.044 min^−1^, 0.067 min^−1^, and 0.114 min^−1^ for reaction temperatures of 20 °C, 30 °C, 40 °C, and 50 °C, respectively. These results clearly demonstrate the strong temperature dependence of the reaction rate and lay the foundation for calculating the apparent activation energy using the Arrhenius equation. The value of *E_a_* for the oxidative degradation of MB using H_2_O_2_ in the presence of NPC-2 was determined using the Arrhenius equation, expressed as follows:

ln*k* = ln*A* − *E_a_*/*RT* (2)

where *κ* is the rate constant, A is the pre-exponential factor, R is the universal gas constant, and T is the absolute temperature. Equation (2) can be simplified to achieve the value of *E_a_* as follows:

(3) $$E_{a}=-R\left[\frac{\partial lnk}{\partial (1/T)}\right]$$

From the slope of the linear fit in the Arrhenius plot (Figure 7c), the apparent activation energy for MB degradation catalyzed by NPC-2 was calculated to be 34.3 kJ mol^−1^. This relatively low activation energy confirms the high intrinsic catalytic activity of NPC-2 and supports the observed temperature-enhanced degradation kinetics. Notably, the *E_a_* value for NPC-2 is substantially lower than that reported for other catalysts, such as ferrocene-based systems, which exhibit an *E_a_* of 82.71 kJ mol^−1^ for MB degradation under similar conditions [65]. This comparison further underscores the efficiency of the nanoporous copper catalyst and its suitability for energy-efficient advanced oxidation processes in wastewater treatment.

### 3.4. Mechanisms of Dye Degradation

The removal of MB from wastewater through green oxidative degradation was further explored by investigating the underlying reaction mechanism in the presence of NPC and H_2_O_2_. Experimental evidence indicates that during the catalytic reaction, highly reactive free radical species, primarily hydroxyl radicals (•OH) and hydroperoxyl radicals (•OOH), are generated from the decomposition of H_2_O_2_ on the surface and within the porous network of NPC. These radicals are responsible for the oxidative breakdown of MB following its adsorption onto the catalyst surface.

**Figure 8:**
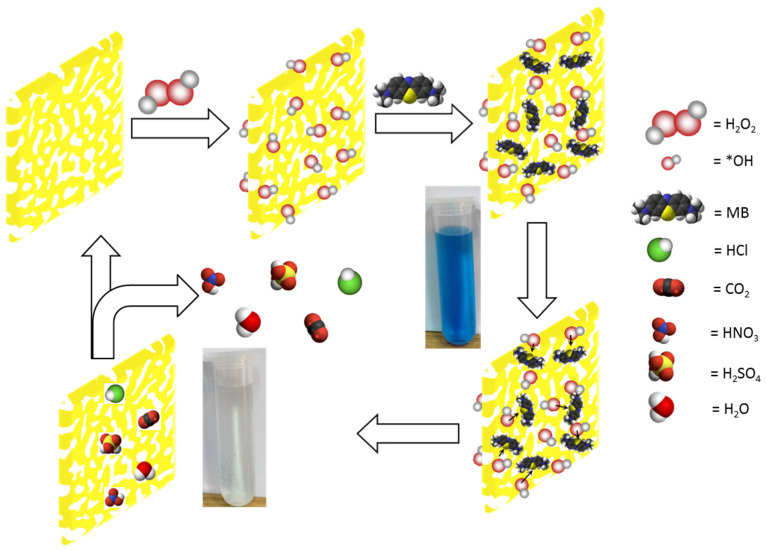
Schematic diagram representing the detailed mechanism of the MB degradation with H_2_O_2_ over NPC based on Langmuir-Hinshelwood model (*OH indicates hydroxyl radicals).

As illustrated in Figure 8, the degradation mechanism proceeds via an adsorption–oxidation–desorption sequence, consistent with the Langmuir–Hinshelwood model [66]. Initially, H_2_O_2_ molecules dissociate on the high-energy active sites of NPC, forming reactive radicals. Simultaneously, MB molecules are adsorbed onto the large internal surface area provided by the bicontinuous ligament–channel network of NPC. Once adsorbed, MB molecules encounter the generated •OH and •OOH radicals, initiating oxidative degradation. This interaction facilitates the breakdown of MB into smaller, non-toxic molecules such as CO_2_, HCl, HNO_3_, H_2_O, and H_2_SO_4_, as previously reported [22,52,67,68,69,70,71,72,73].

Though the XRD and SAED results confirm that the dominant copper phase is metallic Cu^0^, the presence of minor Cu_2_O domains, as suggested by weak SAED rings, may also contribute to the catalytic process. Previous reports have shown that both metallic copper and copper oxides can activate H_2_O_2_, leading to the formation of hydroxyl radicals (•OH) and hydroperoxyl radicals (•OOH) [22,74,75]. In our case, the highly conductive metallic Cu^0^ framework is likely responsible for facilitating rapid electron transfer, while localized oxidized sites may act as additional reactive centers. The synergy between these copper species and H_2_O_2_ promotes the proposed Langmuir–Hinshelwood mechanism for MB degradation.

The efficiency of this process is significantly enhanced in NPC-2 due to its highly uniform and accessible porous architecture, which not only increases the adsorption capacity but also promotes rapid diffusion and effective interaction between MB molecules and reactive species. After oxidation, the resulting degradation products are easily desorbed from the surface and released from the porous network into the solution, completing the catalytic cycle.

This degradation pathway can be described as an adsorption–oxidation–desorption process [76,77], wherein MB molecules first adsorb onto the catalyst surface, undergo oxidation by reactive radical species, and are subsequently desorbed as small, non-toxic byproducts. The overall mechanism aligns well with the Langmuir–Hinshelwood kinetic model [66], which involves surface-mediated reactions between adsorbed reactants and intermediates. This proposed mechanism underscores the vital role of NPC microstructural engineering in facilitating high-efficiency dye degradation and highlights the potential of NPC materials in green environmental remediation technologies.

## 4. Conclusions

In this work, the influence of microstructure on the catalytic performance of NPC for MB degradation was systematically investigated, revealing a strong correlation between alloy composition, porous morphology, and catalytic performance. NPC catalysts with multiscale bicontinuous structures were synthesized via chemical dealloying of Ti–Cu amorphous alloys, with NPC-2 (from Ti_40_Cu_60_) exhibiting a highly uniform ligament–channel architecture. NPC-2 achieved nearly complete MB degradation within 70 min and demonstrated a high pseudo-first-order rate constant of 44 × 10^−3^ min^−1^, far exceeding those of bulk Cu, copper oxide catalysts, and other Cu nanostructures reported in the literature. The apparent activation energy of 34.3 kJ mol^−1^ further confirms the high intrinsic reactivity of NPC-2. The enhanced performance is attributed to the large surface area, high density of active sites, and efficient electron transfer capabilities provided by the nanoporous architecture. The degradation mechanism involves an adsorption–oxidation–desorption pathway governed by the Langmuir–Hinshelwood model, in which MB and H_2_O_2_ molecules are adsorbed onto the porous framework, oxidized by hydroxyl radicals generated in situ, and subsequently desorbed as harmless byproducts. Importantly, this study demonstrates that precise microstructural control is a powerful strategy to tailor the catalytic performance of nanoporous materials. The findings not only provide insights into the structure–property–performance relationship of copper-based catalysts but also offer a scalable and energy-efficient platform for advanced wastewater treatment and broader environmental applications.

## Figures and Tables

**Table 1:** Comparison of our NPC catalyst to other recently reported Cu-based catalysts for oxidative catalytic degradation of MB.

Catalyst Material	Irradiation Light	Degradation Efficiency	Degradation Time (min)	Rate Constant (× 10^−3^ min^−1^)	Ref.
NPC-2	None	98%	70	44	This work
NPC-1	None	95%	100	30	This work
Plate-like CuO	Visible light	97.2%	600	3.7	[55]
CuO-MeOH	Visible light	13%	240	13	[16]
1D-Cu nanoparticles	UV light	84.9%	90	3.78	[56]
Cu-doped silica NPs	–	93.5%	60	7.7	[57]
Copper-based MOFs	Sunlight	100%	120	33	[58]
Cu_0.6_Zn_0.4_Fe_2_O_4_	LED blue light	99.83%	90	34	[59]
WTC-AW-Cu_600_	–	89.3%	90	–	[60]
CuWO_4_ NPs	Sunlight	70%	240	4.5	[61]
CuO/CuS/MnO_2_ NCs	Visible light	98%	160	22	[62]
CuO/Fe_2_O_3_/CuFe_2_O_4_	–	75.5%	120	–	[63]

## Data Availability

The original contributions presented in this study are included in the article. Further inquiries can be directed to the corresponding authors.
